# New insight in lymnaeid snails (Mollusca, Gastropoda) as intermediate hosts of *Fasciola hepatica* (Trematoda, Digenea) in Belgium and Luxembourg

**DOI:** 10.1186/1756-3305-7-66

**Published:** 2014-02-13

**Authors:** Yannick Caron, Koen Martens, Laetitia Lempereur, Claude Saegerman, Bertrand Losson

**Affiliations:** 1 Research Unit in Parasitology and Parasitic Diseases, Department of Infectious and Parasitic Diseases, Faculty of Veterinary Medicine, University of Liège, B-4000 Liège, Belgium; 2 Freshwater Biology, Royal Belgium Institute of Natural Sciences, Brussels, Belgium; 3 Institute of Infection, Immunity & Inflammation, College of Medical, Veterinary & Life Sciences, University of Glasgow, Bearsden Road, Glasgow, G61 1QH Scotland, UK; 4 Research Unit in Epidemiology and Risk Analysis applied to Veterinary Sciences (UREAR-ULg), Department of Infectious and Parasitic Diseases, Faculty of Veterinary Medicine, University of Liège, B-4000 Liège, Belgium

**Keywords:** *Fasciola* sp, *Radix balthica*, *Galba truncatula*, Multiplex PCR, Epidemiology

## Abstract

**Background:**

The present study aims to assess the epidemiological role of different lymnaeid snails as intermediate hosts of the liver fluke *Fasciola hepatica* in Belgium and Luxembourg.

**Methods:**

During summer 2008, 7103 lymnaeid snails were collected from 125 ponds distributed in 5 clusters each including 25 ponds. Each cluster was located in a different biogeographic area of Belgium and Luxembourg. In addition, snails were also collected in sixteen other biotopes considered as temporary wet areas. These snails were identified as *Galba truncatula* (n = 2474) (the main intermediate host of *F. hepatica* in Europe) and *Radix* sp. (n = 4629). Moreover, several biological and non-biological variables were also recorded from the different biotopes. DNA was extracted from each snail collected using Chelex® technique. DNA samples were screened through a multiplex PCR that amplifies lymnaeid internal transcribed spacer 2 gene sequences (500–600 bp) (acting as an internal control) and a 124 bp fragment of repetitive DNA from *Fasciola* sp.

**Results:**

Lymnaeid snails were found in 75 biotopes (53.2%). Thirty individuals of *G. truncatula* (1.31%) and 7 of *Radix* sp. (0.16%) were found to be positive for *Fasciola* sp. The seven positive *Radix* sp. snails all belonged to the species *R. balthica* (Linnaeus, 1758). Classification and regression tree analysis were performed in order to better understand links and relative importance of the different recorded factors. One of the best explanatory variables for the presence/absence of the different snail species seems to be the geographic location, whereas for the infection status of the snails no obvious relationship was linked to the presence of cattle.

**Conclusions:**

Epidemiological implications of these findings and particularly the role of *R. balthica* as an alternative intermediate host in Belgium and Luxembourg were discussed.

## Background

*Fasciola hepatica* is a digenean platyhelminth parasite, also called liver fluke, which induces fasciolosis mainly in domestic ruminants and humans. This disease is responsible for important financial loss on livestock production worldwide. In northern Belgium (Flanders), the yearly cost of infection in dairy production was estimated to be around 8.2 million euros [1], and approximately 52 million euros in Switzerland [2].

Liver fluke needs a lymnaeid snail as intermediate host to complete its life cycle. Although *Galba truncatula* plays this role in Europe [3,4], it seems that other lymnaeid species could also act as alternative intermediate hosts [5]. Globally about 20 species of Lymnaeidae were described as potential intermediate hosts of *Fasciola* spp. [4]. In Europe, several species were experimentally infected or found naturally infected with *F. hepatica*. These species belong to species of the genera *Lymnaea*[6-11]. *Omphiscola*[12-17], *Stagnicola*, *Pseudosuccinea*[18], and *Radix*[6,8,11,16,17,19-22]. These observations suggest that many different species of lymnaeid snails may be potential hosts for the larval stages of *F. hepatica*.

In a previous study, *R. labiata* (Rossmaessler, 1835) was suspected to act as an alternative intermediate host of *F. hepatica* in Belgium since it harbored the intra-molluscan development and allowed subsequent shedding of cercariae. Furthermore, these metacercariae were as infective as those produced in *G. truncatula* as demonstrated by parasitological and serological data collected from experimentally infected rats [20]. Recently, a lymnaeid snail species collected in Ireland was found to be naturally infected by *F. hepatica* and was identified as *R. peregra* by PCR amplification and sequencing of internal transcribed spacer 2 (ITS-2) gene [22].

Systematics of the family Lymnaeidae is controversial as its members can exhibit a great diversity in shell morphology with extremely homogeneous internal anatomical traits [23]. Furthermore, for sibling species belonging to the genus *Radix*, morphometric analyses demonstrated, that shell shape was unsuitable to define homogeneously and discretely recognizable entities, because the variation was continuous [24]. Taxonomy, deduced from rDNA sequences, particularly the ITS-2 may help to differentiate between lymnaeid species [25].

Several techniques aim to detect *Fasciola* sp. in the snail intermediate host such as microscopy or molecular techniques [26]. The latter technique should be effective and cheap enough to screen large numbers of individuals for naturally infected snails. Some studies [22,27-32] used molecular biology - based tools to investigate prevalence of *F. hepatica* in lymnaeids, but very few were tested on naturally infected snails collected in the field.

The aims of the present study were firstly, to assess analytic reliability of DNA extraction and multiplex PCR during a nationwide sampling campaign. Secondly, the potential epidemiological role of other lymnaeid species (*Radix* spp.) was assessed regarding the presence of *F. hepatica* in Belgium and Luxembourg. Finally, the presence and abundance of snails and the prevalence of larval stages of *F. hepatica* in the snail populations were studied in relation with biological and non-biological variables pertaining to the studied ponds.

## Methods

### Sampling

**Figure 1:**
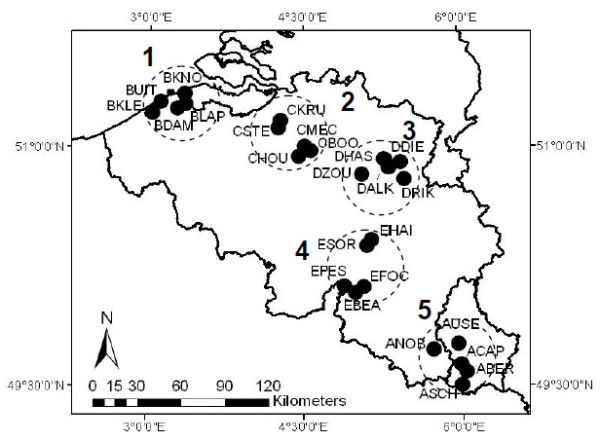
Location of the 25 pond clusters (abbreviations) spread over five biogeographic regions in Belgium/Luxembourg: 1) Polders (BKNO, BUIT, BKLE, BDAM, BLAP), 2) Sand region (CKRU, CSTE, CHOU, CBOO, CMEC), 3) Loam region (DHAS, DZOU, DALK, DRIK, DDIE), 4) Chalk region (ESOR, EPES, EBEA, EFOC, EHAI), 5) Gutland (ANOB, ASCH, ABER, ACAP, AUSE).

Five biogeographic regions were characterized in Belgium and Luxembourg: Polders, Sand region, Loam region, Chalk region and Gutland (Figure 1). In each of these regions, five circular areas of 38 km^2^ were defined, into which 5 ponds were randomly selected. A total of 125 ponds were thus selected according to a strict a priori defined spatial design. Lymnaeid snails (> 4 mm) were sampled in each pond (and surrounding permanent wet area) and additionally in 16 temporary wet areas during summer (July - August) 2008. Snails were collected during a maximum of 15 minutes in spots separated by 4–5 meters all around each pond (irrespective to the presence/absence of snails) and immediately placed in a tube containing 70% alcohol. Several variables were also measured such as GPS coordinates, temperature, pH, soil type (clay, silt, sand, stone), and presence of fence, cattle, trampling, and faecal pats. Ponds were classified in three types: (i) intensively used, mostly characterized by a surrounding environment with high agricultural or breeding of cattle activity, (ii) natural, representing ponds in nature reserves and (iii) extensively used, surrounded by environments with intermediate agricultural or cattle breeding activity. Morphological identification was based on a dichotomous key [33] allowing discrimination between *G. truncatula* and *Radix* sp. using a binocular microscope (x10). Snails were stored in alcohol 70% until further analyses.

### DNA extraction and pooling

DNA extraction was based on Chelex® method as previously described [34]. Briefly, the snail was mechanically disrupted with the help of a pellet mixer (Trefflab) in 100 μl of Chelex® 5% (BioRad) and incubated for one hour at 56°C and 30 min at 95°C in a Peltier Thermal Cycler (MJ Research). The mixture was centrifuged at 13,000 × g for seven minutes. The supernatant was collected and stored at -20°C until further analyses. DNA concentration and quality (260/280 wavelength ratio) were measured using a spectrophotometer (Thermo Scientific, NanoDrop 1000).

In order to reduce the number of PCRs, pools of individuals of the same genus were formed by mixing together one μl of each DNA sample with a maximum of 10 snails per pool. This mixture was considered undiluted. One μl of the mixture was then tested in the multiplex PCR described below.

The absence of internal control amplification (PCR inhibitors) for a pooled or an individual sample was assessed through 1/10 and 1/100 dilutions. Furthermore, the addition of 0.05% Bovine Serum Albumin (BSA) in the PCR mixture at 1/10 dilution was tested for samples with absence of internal control amplification whereupon negative samples were definitely excluded from the study.

### Multiplex PCR and sequencing reaction

A multiplex PCR assay [34] was used to amplify a highly repeated 124 bp sequence (microsatellite) specific for *Fasciola* spp. [35] and ITS-2 rDNA sequence specific for lymnaeids (500–600 bp). ITS-2 sequence of the snail acts as a PCR internal control as its absence indicates potential presence of PCR inhibitors. The primers used for amplification of *Fasciola* spp. sequences were Fsh1 5′-GAT-CAA-TTC-ACC-CAT-TTC-CGT-TAG-TCC-TAC-3′ and Fsh2 5′-AAA-CTG-GGC-TTA-AAC-GGC-GTC-CTA-CGG-GCA-3′ and for lymnaeids ITS-2 amplification were News2 5′-TGT-GTC-GAT-GAA-GAA-CGC-AG-3′ and Its2Rixo 5′-TTC-TAT-GCT-TAA-ATT-CAG-GGG-3′ [36,37]. The sequences were amplified using a commercial kit (Taq PCR Master Mix, Qiagen) in a total volume of 25 μl in a Peltier Thermal Cycler (MJ Research) with an initial denaturation step at 95°C for five minutes, followed by 40 cycles, each comprising denaturation at 95°C for one minute, annealing at 56°C for one minute, extension at 72°C for one minute and a final extension step at 72°C for ten minutes. The amplification products were electrophoretically resolved in 2% agarose gels and stained with ethidium bromide. The limit of detection and specificity of this multiplex PCR were studied in a previous study [34].

ITS-2 rDNA sequence of 100 *Radix* sp. (93 randomly selected and 7 *Fasciola* sp. positive samples) were amplified and sequenced for species identification. ITS-2 DNA products were purified using MSB-Spin PCRapace (Invitek). Cycle sequencing reactions were performed (in duplicate and in both direction) by BigDye terminator v3.1 (3730 DNA analyzer; Applied Biosystems) by GIGA Genomics Facility (Liège University, Belgium). Consensus sequences were made according to the results of sequencing of the PCR products and were analyzed using BLASTn searches in GenBank (http://www.ncbi.nlm.nih.gov) and aligned using BioEdit 7.0.9.0 [38].

### Statistical analysis

#### Classification and regression tree (CART) analysis

A CART analysis is a non-linear and non-parametric model that is fitted by binary recursive partitioning of multidimensional covariate space [39]. Using CART 6.0 software (Salford Systems, San Diego, CA, USA), the analysis successively splits the dataset into increasingly homogeneous subsets until it is stratified and meets specified criteria. CART performs cross validation by growing maximal trees on subsets of data then calculating error rates based on unused portions of the data set.

When the primary splitting variable is missing for an individual observation, that observation is not discarded but, instead, a surrogate splitting variable is sought. Thus, the program uses the best available information in the face of missing values. In datasets of reasonable quality, this allows all observations to be used. This is a significant advantage of this methodology over more traditional multivariate regression modelling, in which observations that are missing *any* of the predictor variables are often discarded. Further details about CART are presented in previously original papers or reviews [40,41].

A CART analysis was conducted on two data sets. Four different analyses were performed. For CART I and II, the dependant variable was the presence of *Radix* sp. or *G. truncatula*, respectively and the independent variables were the county of origin of the pond, geologic characteristics of the soil, type and depth of pond, and temperature and pH of the water. For CART III and IV, the dependant variable was the presence of infected *Radix* sp. or *G. truncatula* in ponds, respectively and the independent variables were the presence of fences, (traces of) presence of animals, the type of pond and the number of specimens of *Radix* sp. or *G. truncatula.*

#### Assessment of lymnaeid presence data

The presence of *Radix* sp. and *G. truncatula* in all the biotopes were compared in order to assess their agreement with our results using Fisher’s exact test [42] and concordance analysis. The level of agreement was also expressed in terms of indices of positive and negative agreement [43], respectively the observed agreement proportion for positive and negative results (i.e., presence and absence).

## Results

### Sample collection

**Figure 2:**
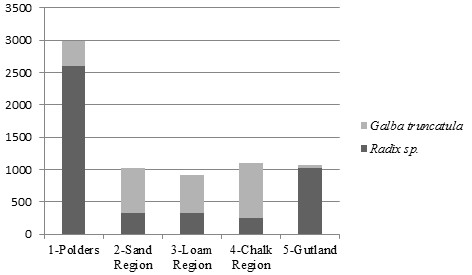
**Number of *G. truncatula *** **and *Radix *** **sp. found in the five biogeographic regions.**

Seven thousand one hundred and three lymnaeid snails were collected during summer 2008 of which 2474 were morphologically identified as G. *truncatula* (34.8%) and 4629 as *Radix* sp. (65.2%) (Figure 2). Lymnaeid snails were found in 53.2% (75/141) of biotopes investigated, in which *G. truncatula* was found in 60% (45/75) of all the biotopes and 80% (36/45) in ponds, whereas *Radix* sp. was recorded in 64% (48/75) of all the biotopes and 85.4% (41/48) in ponds. A percent of 35.8% (886/2474) and 8.2% (378/4629) of the collected *G. truncatula* and *Radix* sp. were collected from the sixteen temporary wet areas respectively. *G. truncatula* and *Radix* sp. were present together in 24% (18/75) of the biotopes. *G. truncatula* was most abundant in Sand, Loam and Chalk Regions (83.2%). Most of the *Radix* species were found in Polders (57.2%), where *G. truncatula* were relatively under-represented.

### Multiplex PCR and sequencing

Two thousand four hundred and seventy four *G. truncatula* were pooled in 271 batches. Although different dilution factors were tested (undiluted, 1/10, 1/100 and 1/10 + BSA), 195 snails (7.89%) were eliminated from the study due to PCR inhibition. Twenty eight pools containing 270 snails were found positive for *Fasciola* sp. These 270 snails were individually screened using the multiplex PCR and 30 *G. truncatula* were found positive, resulting in a prevalence of 1.31% (30/2279).

**Figure 3:**
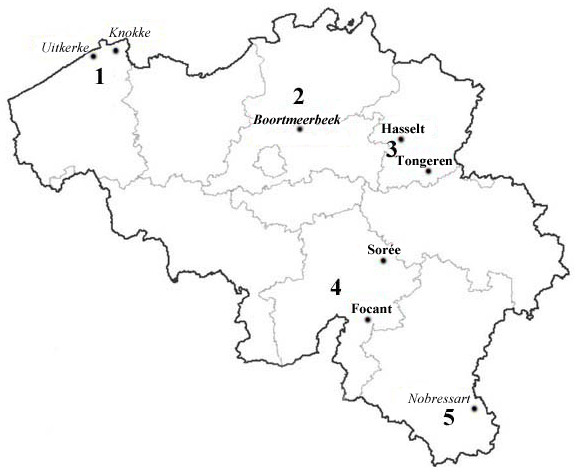
**Location of the ponds where *Fasciola *** **sp. infected snails species were found: 1) Polders, 2) Sand region, 3) Loam region, 4) Chalk region, 5) Gutland; Bold for *G. truncatula*; Italic for *Radix *** **sp.; Italic and bold for pond where *G. truncatula *** **and *Radix *** **sp. were found infected.**

Four thousand six hundred and twenty nine *Radix* sp. were pooled in 486 batches of a maximum of 10 snails each. Four hundred and four snails (8.77%) were eliminated from the study due to PCR inhibition. The 124 bp specific band of *Fasciola* sp was amplified in six pools (60 snails). These 60 snails were individually tested and 7 snails were positive. The frequency of snails with parasite DNA in *Radix* sp. was estimated to be 0.16% (7/4225). The geographical location of ponds where *Fasciola* sp. infected snails was found is illustrated in Figure 3.

In order to accurately identify *Radix* sp., ITS-2 of the 7 *Fasciola* sp. DNA positive *Radix* sp. and 93 other *Radix* sp. randomly selected were sequenced. Ninety eight sequences (named as PRT1) [GenBank: KC544264] were found to be 100% identical to *R. peregra* (Müller, 1774) (GenBank: HQ283271.1). The two remaining sequences (named as PRT2) [GenBank: KC544265] were identified as 100% identical to *R. labiata* (Rossmaessler, 1835) [GenBank AJ319637.1]. The 7 *Radix* sp. positive for *Fasciola* sp. all belonged to PRT1.

### Statistical analysis

#### CART analysis

##### Presence/absence

The presence/absence of *G. truncatula* (CART I) and *Radix* sp. (CART II) were assessed for the 125 sampled ponds using CART analysis. Table 1 presents results of both CART analyses. The geographic location seems to be the best explanatory variable for the presence of *G. truncatula* and *Radix* sp. Presence of *G. truncatula* seems strongly linked to pH, water temperature and ground type whereas presence of *Radix* sp. is essentially related to the ground type.

**Table 1:** **CART analysis I ( *G. truncatula *) and II ( *R. balthica *): presence/absence; explanatory percentage for each of the independent variables was indicated**

***G. truncatula***		***R. balthica***	
County	100%	County	100%
pH	73%	Ground type	59.25%
Water temperature	46.81%	Pond type	23.04%
Ground type	45.32%	Water temperature	23.04%
Pond type	13.85%	pH	14.25%
Tree sensibility	84.44%	Tree sensibility	81.25%
Tree sensitivity	81.25%	Tree sensitivity	60.22%

##### Infected/non infected

The infection status of *G. truncatula* (CART III) and *Radix* sp. (CART IV) was also assessed using CART analysis. Table 2 presents results of both CART analyses. The number of collected snails was identified as the best explanatory factor. Factors relative to cattle presence (trampling, faecal pats, and fence) was not linked to the infection status.

**Table 2:** **CART analysis II ( *G. truncatula *) and IV ( *R. balthica *): infection status; explanatory percentage for each of the independent variables was indicated**

***G. truncatula***		***R. peregra***	
*Number of snail*	*100%*	*Number of snail*	*100%*
*Pond type*	*22.14%*	*Ground type*	*29.84%*
Tree sensibility	83.33%	Tree sensibility	100%
Tree sensitivity	97.78%	Tree sensitivity	82.35%

#### Assessment of lymnaeid presence data

The distribution of *G. truncatula* and *Radix* sp. was not significantly different within biotopes (Fischer’s exact test; *P* = 0.34). The P_pos_ and P_neg_ are 0.39 and 0.70, respectively.

## Discussion

This is the first time a molecular based technique has been used to assess natural *Fasciola* sp. prevalence in intermediate hosts in Belgium. One hundred and twenty five ponds and sixteen other interesting areas in five different biogeographic regions in Belgium and Luxembourg were sampled for snails with environmental factors information. In Belgium in 2008, winter and spring were particularly mild. Furthermore, during that summer the rainfall and the number of rainy days were quite abnormal (Royal Meteorological Institute of Belgium; http://www.meteo.be). Those conditions are the most suitable for snail development. More than half of these biotopes (53.2%) were colonized with lymnaeid snails. Soil composition in Flanders (sand, silt) in Polders, Sand Region, and Loam region seem to be suitable for the development of lymnaeid snails. A great diversity in shell morphology with extremely homogenous anatomical traits created a great confusion regarding the systematics of lymnaeids especially those belonging to the genus *Radix*. Some authors [25] considered *R. peregra* (Müller, 1774) as a synomym of *R. ovata* (Draparnaud, 1805) and *R. balthica* (Linnaeus, 1758), while others maintained all species as valid [44]. In this present study, *R. balthica* was used to represent all of these synonymous species (*R. peregra* = *R. ovata* = *R. balthica* = PRT1) because the name *R. peregra* was abandoned by Bargues *et al.*[37]. The genus *Radix* was more represented than the main intermediate host of *F. hepatica* since only one third of the 7103 collected snails belonged to the species *G. truncatula*. Indeed, *R. balthica* (= *R. peregra* = *R. ovata*) is considered as a very widespread and resilient species [45], which is able to live in waste water [46], in contrast to *G. truncatula*. Furthermore, ponds are not typical biotopes of *G. truncatula*, which prefers peripheral extremities of open drainage furrows, spring head surroundings, temporary wet meadow, and ditches [47]. In sixteen of those specific biotopes, *G. truncatula* (35.8%) was more frequent than *Radix* sp. (8.2%). However, the Fisher’s exact test indicates that there is no difference between lymnaeid fauna composition and biotopes (permanent wet vs temporary wet areas). Prevalence of *F. hepatica* in permanent and temporary water habitats were never compared even though some consider these as equal [48].

Establishment of pools including a maximum of 10 snails was advantageous to reduce PCR number and consequently cost and time consumption. The proportion of PCR inhibition was relatively high despite dilution and use of BSA [27,34]. More than 66% (130/195) of the discarded *G. truncatula* snails were collected in a tractor track probably polluted with some PCR inhibitors (complex polysaccharide, humic acid, or proteinase).

*Fasciola* sp*.* prevalence was evaluated to 1.31% in *G. truncatula* and 0.16% in *Radix* sp using an optimized multiplex PCR. This PCR-based prevalence is relatively low in the main intermediate host of liver fluke in Europe, particularly when compared to prevalence obtained with microscopy-based techniques [26]. This could be due to the pooling process, which is responsible for a dilution of PCR available DNA (up to 1/100). However, in a previous study [34] a pool containing the DNA from one *F. hepatica* naturally infected snail and 9 negative specimens, was found positive using the same technique (total DNA concentration of 100 pg (1/10.000 dilution)). Only two studies assessed the prevalence of *Fasciola* sp. in naturally infested *G. truncatula* with PCR-based techniques. In Poland, a preliminary study using a limited number of snails (<200) and based on the same 124 bp repeated sequence [29] estimated the prevalence to be 26.2%. In Switzerland [32] more than 4700 snails were collected with a prevalence estimated to be 7% using a real time TaqMan PCR. Nevertheless, these snails were collected in infected cattle farms and the real regional prevalence was probably overestimated.

*Fasciola* sp. infection rates in other intermediate hosts vary greatly from less than 1% to more than 60%: 0.032% (1/3072) in *L. modicella*[31]; 1.5% (79/5246) in *Fossaria cubensis*[28]; 51.3% (123/240) in *L. columella* and 61.8% (21/34) in *L. viatrix*[27]. In Ireland, a study [22] using PCR amplifying a part of the cytochrome c oxidase subunit 1 (cox 1) gene provided prevalence of 73.9% (n = 17) in *Succinea* sp. and from 10.3% (n = 8) to 61.1% (n = 22) in *R. balthica* (= *R. peregra* = *R. ovata*). *Radix balthica* may harbour incidental infection and experimental infections are difficult to implement [49,50]. However, other authors have shown [20-22,25] that *R. balthica* can maintain intermolluscan stages of *F. hepatica* enabling the parasite to multiply. The frequency of snails with parasite DNA obtained for *R. balthica* in this study was very low and its epidemiological role seems weak. Nevertheless, *R. balthica* (= *R. peregra* = *R. ovata*) are more aquatic and more invasive [51] than amphibious *G. truncatula* and could lead to the extension of fasciolosis in previously free areas, particularly where *G. truncatula* is absent [6,13,22]. The genus *Radix* seems to be very permissive to Trematoda infection as natural infections were recorded for *F. gigantica*[52]*Fascioloides magna*[51], *Trichobilharzia regenti*[53,54], and *T. franki*[55], and finally echinotostomatid species [37]. The reasons for this parasitic tolerance are poorly understood although widespread distribution, high density and immune mechanisms of this genus seem to be the most plausible hypothesis. Studies on niche modeling predicted northward expansion of *R. balthica* (= *R. peregra* = *R. ovata*), as already observed in Sweden probably due to increasing water temperature in lakes (M. Pfenninger, unpublished data). During a previous study [20], *R. labiata* (Rossmaessler, 1835) (= PRT2) was shown to be able to shed cercariae. The metacercariae obtained were infective to rats in the laboratory contrary to metacercariae obtained from experimentally infected *R. balthica.* This could be linked to the difficulties encountered during breeding of this last species under laboratory conditions (highest average mortality: 54%) [20] or prevalence variability between populations as it was described for *G. truncatula*[56]. Furthermore this species was under-represented in the samples.

The presence/absence of lymnaeids and their infestation status were both assessed using CART analysis. *Galba truncatula* seems to be more stenoecious, since this species is very sensitive to environmental factors (pH and water temperature for example) and lives in a more restricted range of habitats [47] than *R. balthica* (the “travelling species”) which is more tolerant to variable environmental conditions.

The number of snails collected was found as the best explanatory factor related to the infection status. Surprisingly, presence of cattle (trampling, stools, fences) was not found to be a factor linked to the snail infestation status. However, a lack of power in the analyses is possible, because of the low prevalence of infection observed and this might have caused non-significant correlations. This could also suggest that wild fauna can intervene in the maintenance of *F. hepatica* life cycle. Generally, farmers consider that the presence of ponds in their meadow has a negative impact on cattle breeding, while European funds support the maintenance and digging of ponds in order to promote biodiversity. This last result could highlight the importance of temporary aquatic biotopes in the epidemiology of *F. hepatica* in Belgium. Nevertheless, pond types (intensive, natural and extensive) were not very informative on the infestation status (less than 30% in Cart analysis Table 2).

Digenetic trematode species are usually oioxenous (one parasite species infect a single snail species) or stenoxenous (one parasite species infect several closely related snail species). But the case of *F. hepatica* seems to be different as it shows a broad capacity to infect local and phylogenetically distant lymnaeid species [44]. Therefore, rather than focusing on a single, or a handful of snail species, fasciolosis control programs should cover a broader spectrum of intermediate hosts that inhabit diverse habitats and ecological conditions [44]. The multiplex PCR method used in this study confirms this approach can address such concerns and highlights the role of *R. balthica* (= *R. peregra* = *R. ovata*) in the epidemiology of *F. hepatica* in Belgium.

## Conclusion

A multiplex PCR was used to assess the parasitological status of 7103 lymnaeid snails collected in natural biotopes in Belgium. This technique was fully reliable and this is the first time in Belgium that naturally infected snails collected in the field were analyzed through molecular biology – based tools.

## Competing interests

The authors declare that they have no competing interests.

## Authors’ contributions

YC wrote the manuscript and made sampling and analysis. KM managed the PONDSCAPE project and participated in the reviewing process. LL participated in the reviewing process. CS made statistical analysis. BL supervised the whole process. All authors read and approved the final version of the manuscript.
